# Effect of *Mucuna pruriens* seed extract on depression-like behavior derived from mild traumatic brain injury in rats

**DOI:** 10.37796/2211-8039.1461

**Published:** 2024-09-01

**Authors:** Alfonso Mata-Bermudez, Ricardo Trejo-Chávez, Marina Martínez-Vargas, Adán Pérez-Arredondo, María de los Ángeles Martínez-Cardenas, Araceli Diaz-Ruiz, Camilo Rios, Héctor A. Romero-Sánchez, Agustino Martínez-Antonio, Luz Navarro

**Affiliations:** a Departamento de Fisiología Facultad de Medicina, Universidad Nacional Autónoma de México, Apdo.Postal 70-250, 04510, Ciudad de México, Mexico; b Doctorado en Ciencias Biomedicas, Universidad Nacional Autónoma de México, Apdo. Postal 70-250, 04510, Ciudad de México, Mexico; c Departamento de Atención a la Salud, Universidad Autónoma Metropolitana Unidad Xochimilco, Ciudad de México, Mexico; d Departamento de Neuroquímica, Instituto Nacional de Neurología y Neurocirugía Manuel Velasco Suarez, Ciudad de México, Mexico; e Laboratorio de Neurofarmacología Molecular, Departamento de Sistemas Biológicos, Universidad Autónoma Metropolitana Unidad Xochimilco, Ciudad de México, Mexico; f Dirección de Investigación. Instituto Nacional de Rehabilitación Luis Guillermo Ibarra Ibarra, Ciudad de México, Mexico; g Biological Engineering Laboratory, Genetic Engineering Department, Center for Research and Advanced Studies of the National Polytechnic Institute (Cinvestav), Campus Irapuato, Guanajuato, Mexico

**Keywords:** *Mucuna pruriens*, Depression, Traumatic brain injury, Oxidative stress, Lipid peroxidation, Reduced glutathione

## Abstract

**Background:**

Traumatic brain injury (TBI) is a severe health problem for which there is no specific treatment, leading to neurological or neuropsychological consequences. One of the most described disorders, even after mild TBI (mTBI), is depression, related to mechanisms involving reactive oxygen species (ROS). The *Mucuna pruriens* (*M. pruriens*) plant has various antioxidant, neuroprotective, and anti-inflammatory properties.

**Purpose:**

There is insufficient evidence of *M. pruriens* use for the treatment of neurobehavioral and depressive impairments induced by TBI and of the mechanisms underlying this effect, so we aimed to evaluate the ability of shortterm administration of *M. pruriens* extract to prevent neurobehavioral impairment and depression-like behaviors in a murine model of mTBI as well as evaluate the role of oxidative stress.

**Methods:**

Male Wistar rats underwent mTBI or sham surgery. Immediately after, they were treated with vehicle or *M. pruriens* extract (50 mg/kg ip/day for five days). We evaluated neurobehavioral recovery using the Neurobehavioral Severity Scale-Revised (NSS-R) and the immobility time in the forced swimming test 3, 7, 15, 30, and 60 days after mTBI. In addition, lipid peroxidation (LP) and GSH concentrations were determined in some brain areas (motor cortex, striatum, midbrain, and nucleus accumbens).

**Results:**

*M. pruriens* extract did not decrease neurobehavioral impairment caused by mTBI. Nevertheless, it prevented depression-like behaviors starting three days after mTBI, reduced LP, and increased GSH in some brain areas. *Conclusions*: *M. pruriens* may prevent depression-like behaviors and reduce oxidative stress by decreasing LP and increasing concentrations of antioxidant compounds.

## 1. Introduction

Traumatic brain injury (TBI) is defined as damage caused by an external mechanical force that induces disruption of brain function or other evidence of pathology [1]. TBI represents one of the leading causes of chronic disability since it affects approximately 69 million people worldwide yearly [2]. Mild TBI (mTBI) is the most prevalent, with approximately 55 million cases annually [2]. Among TBI’s most common and difficult-to-treat long-term psychiatric complications are depressive disorders, with an estimated prevalence of 20%–45% [3], even after mTBI [4]. Depression is a mood disorder that causes persistent sadness, apathy, and an inability to enjoy everyday life events [5]. It is worth noting that although serotonin reuptake inhibitors are the first line of treatment to control depressive disorders [3], the exact etiopathogenesis of depression is still not well understood. In addition to the monoaminergic imbalance [6], depression has been associated with a disproportion between the production of reactive oxygen species (ROS) and the antioxidant defenses [7]. More recently, an inflammatory theory of depression has been proposed. About it, various research has reported that the activation of the immune system plays an essential role in the development of depression. In particular, they have suggested that tumor necrosis factor alpha (TNF-α) and some proinflammatory interleukins have been associated with the pathogenesis of depression [8,9].

*Mucuna pruriens (M. pruriens)*, commonly known as the velvet bean, has anti-inflammatory, antiepileptic, antimicrobial, and antioxidant properties and has been used to treat Parkinson’s disease [10,11]. *M. pruriens* extracts decrease various markers of oxidative stress and increase the activity of brain antioxidant enzymes in animal models of Parkinson’s disease [12] and chronic treatment with *M. pruriens* and its main component L-3,4-dihydroxyphenylalanine (L-Dopa) decreased ROS levels in several experimental models, for example, in testicular germ cells [13]. *M. pruriens* seeds significantly reduced depressive-type behaviors in animal models, such as the duration of immobility in the forced swimming and tail suspension tests [14]. Likewise, the anti-inflammatory effect of *M. pruriens* has been reported in Parkinson’s models [15]. In particular, it has been shown to prevent the increase in TNF induced by the neurotoxin MPP^+^ or lipopolysaccharide (LPS) [16]. All these findings suggest that treatment with *M. pruriens* could represent a pharmacological alternative for treating depression derived from mTBI. In this work, we evaluate the short-term administration of the lyophilized extract of *M. pruriens* in neurobehavioral deterioration, the development of depression-like behaviors in rats with mTBI, and the ROS modulation in these effects.

## 2. Methods

### 2.1. Experimental subjects

The experiments were carried out with adult male Wistar rats (250–300 g). The animals were acclimated to standard laboratory conditions (12-h light–dark cycle, 23 ± 1 °C, lights on at 10:00 AM) with free access to water and food for seven days. All experimental procedures were approved by the Ethics Committee of the Faculty of Medicine of the National Autonomous University of Mexico (UNAM) (Project: 031-CIC-2019) and followed the national and international guidelines of the Official Mexican Standards NOM-062-ZOO-1999 and the Guidelines for the Care and Use of Laboratory Animals from the National Institutes of Health (USA). The animals were provided by the Animal Production Unit-Bioterio (Bioterio) of the Faculty of Medicine of the UNAM.

Lyophilized extract of the *M. pruriens* plant seeds is enriched in L-Dopa (56%) [17]. It was provided by Biofab México (Irapuato, Guanajuato, MEX). The extract was dissolved for administration in normal saline solution (NS) to use a dose of 50 mg/kg (containing 28 mg/kg of L-DOPA) as suggested by Wang et al., [18].

### 2.2. Experimental design

Two hundred ten male Wistar rats (250–300 g) were used, distributed in 20 experimental groups. Fifteen minutes after mTBI, ten groups received a dose of the lyophilized extract of the *M. pruriens* plant (50 mg/kg, i.p.) or the vehicle (NS) once daily for five days. Control groups included healthy animals without manipulation (naïve, N) and animals with a simulated surgical procedure (sham, S). Motor and sensory disturbances were assessed using the Neurobehavioral Severity ScaleRevised (NSS-R) [19], and depression-like behaviors were assessed using the forced swimming test [20]; these behaviors were monitored 3, 7, 15, 30, and 60 days after mTBI. At the end of the experiments, the animals were sacrificed by decapitation (after undergoing anesthesia with sodium pentobarbital, 50 mg/kg, i.p.). Brain regions (motor cortex, striatum, midbrain, and nucleus accumbens) were extracted to determine the LP and quantify the GSH concentration.

### 2.3. Surgical procedure

Animals were anesthetized with a mixture of ketamine (66 mg/kg, i.p.), xylazine (0.26 mg/kg, i.p.), and acepromazine (1.3 mg/kg, i.p.) [21]. The animals were placed individually in a stereotaxic apparatus. An incision of approximately 2 cm in diameter was made along the midline to expose the skull. Methods used to establish a closed head injury TBI model were previously standardized in our laboratory [21]. Briefly, the exposed skull of the animal was impacted by a 1 mm diameter pneumatic piston (20 lb., 4 mm deep) at anterior-posterior (AP) and lateral (L) stereotaxic coordinates of −2 mm and +1.4 mm, respectively, which corresponds to the primary motor cortex (M1).

### 2.4. Neurobehavioral Severity Scale-Revised (NSS-R)

The motor and sensory responses were evaluated using the NSS-R [19]. This scale includes ten tasks that assess balance, motor coordination, and sensorimotor reflexes. For each task, a numerical evaluation rank is used in which 0 represents optimal neurological status, 1 corresponds to a partial or compromised response, and 2 indicates that the study subject could not perform the task [19].

### 2.5. Forced swimming test

Animals were placed individually for 10 min in a transparent acrylic cylinder 20 cm in diameter and 50 cm high; tap water was added at a temperature of 23 ± 2 °C until it reached a height of 30 cm; the duration of immobility by the rat was measured, defined as the absence of any movement, except that necessary to keep the head out of the water [20].

### 2.6. Lipid peroxidation (LP)

Samples of injured brain tissue were used to measure fluorescent products of LP. Tissue samples were homogenized in 3 mL of NS. One milliliter of homogenate was added to 4 mL of a chloroformmethanol mixture (2:1, v/v). After stirring, the mixture was chilled with ice over 30-min intervals to allow phase separation. The fluorescence emitted from the chloroform phase was measured at 370 nm (excitation) and 430 nm (emission). Quinine standard solution (0.1 g/mL) was used to adjust the sensitivity of the spectrophotometer to 150 fluorescence units. The results are expressed as fluorescence units/g tissue [22].

### 2.7. Reduced glutathione (GSH)

The concentration of GSH in brain tissues was determined, as described by Baron-Flores (2022) [22]. GSH standard was prepared daily in 0.1 M sodium phosphate with 5 mM EDTA buffer (pH 8). The o-phthalaldehyde (OPA) solution was prepared in reagent-grade absolute methanol. Samples were homogenized in 3.75 mL of EDTAphosphate buffer (pH 8.0) plus 1 mL of HPO_3_ (25%). The homogenates were centrifuged at 3000×*g* for 15 min, and the supernatants were separated. 500 ul of supernatant was added to 4.5 mL of phosphate buffer plus 100 μL of OPA and incubated at room temperature for 15 min. Fluorescent signals were measured at 350 nm excitation and 420 nm emission wavelengths. The results were expressed as moles of glutathione per gram of wet tissue [22].

### 2.8. Statistical analysis

The results are presented as the mean ± standard deviation. Significant differences among the groups were determined using a two-way analysis of variance followed by the two-stage linear step-up procedure of Benjamin, Krieger, and Yekutieli for continuous and normally distributed variables or by a Kruskal–Wallis test followed by the Mann–Whitney U test for discrete variables, using Graph-Pad Prism version 9.3.1 for Windows.

## 3. Results

### 3.1. Neurobehavioral responses

**Fig. 1:**
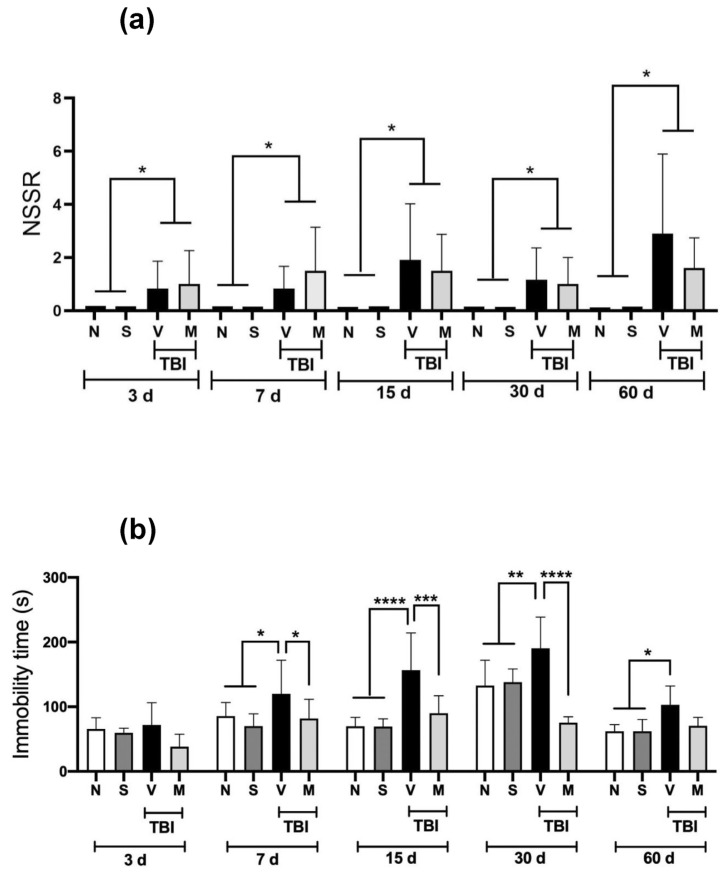
Effect of short-term treatment with the lyophilized extract of the M. pruriens plant on behavioral tests in rats with a mTBI. The time course of short-term administration (once a day for five days) of the lyophilized extract of the M. pruriens plant (50 mg/kg, i.p.) on (a) the score obtained in the Neurobehavioral Severity ScaleRevised (NSS-R) and (b) the immobility duration in the forced swimming test shown in naïve rats (N), sham-operated rats (S) and rats with mTBI that received the vehicle (V) or M. pruriens extract (M) evaluated at 3, 7, 15, 30 and 60 days after the injury. Values are the mean ± standard deviation of 6–12 animals per group. *p ≤ 0.05 according to Kruskal–Wallis and Mann–Whitney U tests for NSS-R values or *p ≤ 0.05; **p < 0.01; ***p < 0.001; ****p < 0.0001 according to two-way ANOVAs followed by the two-stage linear step-up procedure of Benjamini, Krieger, and Yekutieli for immobility time.

The neurobehavioral response to short-term treatment (15 min after mTBI and once a day for five days) with the lyophilized extract of the *M. pruriens* plant is shown in Fig. 1a. The (naïve, N) and the rats that underwent a sham surgical procedure (sham, S) obtained a score of zero in all the periods analyzed, indicating that they were in an optimum neurological state. In contrast, rats subjected to mTBI and administered vehicle (V) exhibited a significant increase (p < 0.05) in NSS-R scores in all the periods analyzed. The short-term administration of the lyophilized extract of the *M. pruriens* plant (50 mg/kg, i.p.) failed to reverse this behavioral deterioration significantly.

### 3.2. Evaluation of depressive-type behavior

Fig. 1b shows the immobility duration values in the forced swimming test, which was used to assess depressive-type behavior. mTBI induced a significant increase in immobility duration from Day 7 post-TBI to 60 days post-TBI compared to the N and S groups. The administration of the lyophilized extract of the *M. pruriens* (M) plant prevented this increase on Days 7–30 after mTBI. On Day 60 post-mTBI, the immobility duration of M rats was not significantly different from that of the V group.

### 3.3. Evaluation antioxidant effect

**Fig. 2:**
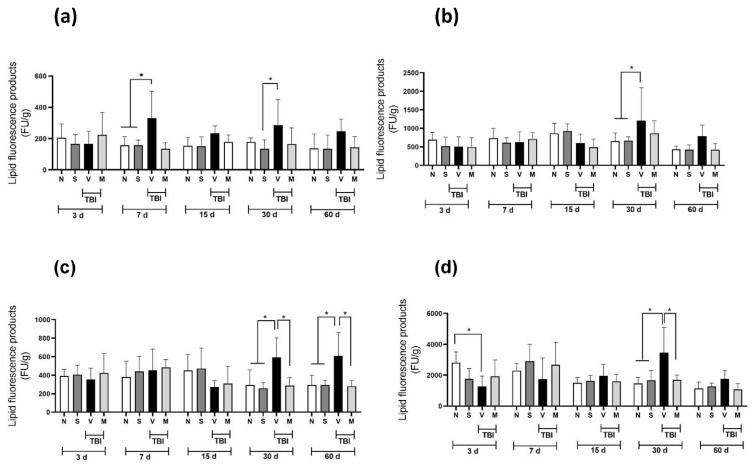
Effect of short-term treatment with the lyophilized extract of the M. pruriens plant on lipid peroxidation concentrations in injured tissue in rats with mTBI. The lipid peroxidation concentration in the motor cortex (a); striatum nucleus (b); midbrain (c); and nucleus accumbens (d) are shown in naïve rats (N), sham-operated rats (S), and rats with mTBI that received the vehicle (V) or the lyophilized extract of the M. pruriens plant (50 mg/kg, i.p., once a day for five days) (M) 3, 7, 15, 30 and 60 days after mTBI. The values are expressed as the mean ± standard deviation of 6 animals per group. *p ≤ 0.05 according to two-way ANOVAs followed by the two-stage linear step-up procedure of Benjamini, Krieger, and Yekutieli.

**Fig. 3:**
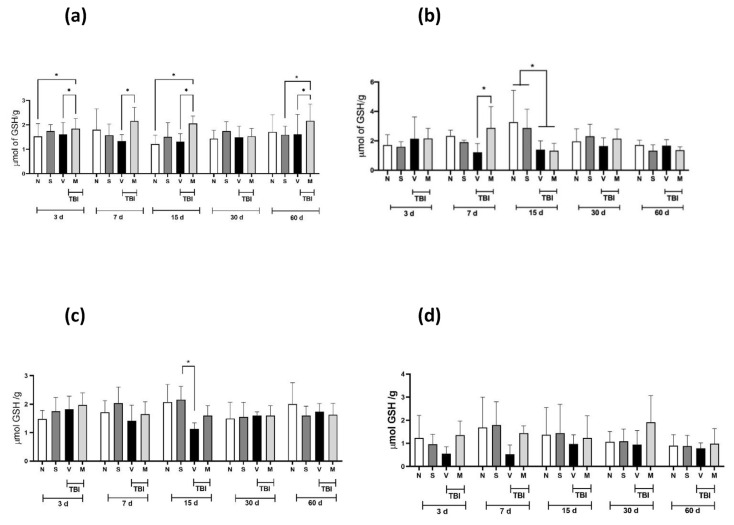
Effect of short-term treatment with the lyophilized extract of the M. pruriens plant on the concentration of GSH present in the injured tissue in rats with mTBI. GSH concentrations are shown in the motor cortex (a); striatum nucleus (b); midbrain (c); and nucleus accumbens (d) in naïve rats (N), sham-operated rats (S), and mTBI rats that received the vehicle (V) or the lyophilized extract of the M. pruriens plant (50 mg/kg, i.p., once a day for five days) (M) at 3, 7, 15, 30 and 60 days after mTBI. The values are expressed as the mean ± standard deviation of 6 animals per group. *p ≤ 0.05 according to two-way ANOVAs followed by the two-stage linear step-up procedure of Benjamini, Krieger, and Yekutieli.

Fig. 2 shows the LP values obtained in four different brain regions from rats with mTBI: the motor cortex (a), striatum (b), midbrain (c), and nucleus accumbens (d). mTBI induced a significant increase (p < 0.05) in LP concentration in the motor cortex (Fig. 2a) on Day 7 and Day 30 post-mTBI; on Days 15 and 60, these values did not reach statistical significance. In the striatum (Fig. 2b), a significant increase in LP concentration was observed only in the TBI + V group compared to the N and S groups on Day 30. In the midbrain (Fig. 2c), mTBI induced a significant increase in LP concentrations on Days 30 and 60 in the V group compared with the N and S groups; short-term administration of the lyophilized extract of the *M. pruriens* plant prevented this increase. Regarding the nucleus accumbens (Fig. 2d), the increase in LP concentrations induced by mTBI was only observed on Day 30; this increase was prevented by short-term administration of the lyophilized extract of the *M. pruriens* plant. Fig. 3 shows the GSH values in four different brain regions of rats with mTBI: the motor cortex (a), striatum (b), midbrain (c), and nucleus accumbens (d). For the motor cortex (Fig. 3a), on almost all of the days analyzed, short-term administration of the lyophilized extract of the *M. pruriens* (M) plant significantly increased the concentration of GSH compared to the V group. In the striatum (Fig. 3b), this increase in GSH concentration was observed only on Day 7, while on Day 15, a decrease in GSH concentration was observed. Regarding the midbrain (Fig. 3c), we observed that mTBI decreased the concentration of GSH compared to the S group on Day 15 only. In contrast, in the nucleus accumbens, we did not observe any statistically significant difference (Fig. 3d).

## 4. Discussion

Using a mTBI model, we found (Fig. 1a) an impaired motor and sensory function in rats three days to 60 days after the injury, using the NSS-R. Although most authors have reported transitory motor and cognitive deficits using mTBI models [23], several authors agree that various deficits persist for several weeks and up to a year post-TBI [24].

Due to the limitations of conventional therapies and the lack of specific treatments for TBI, interest in the study of the medicinal properties of natural products has increased considerably due to their beneficial properties. *M. pruriens* extracts possess antioxidant and anti-inflammatory properties and have been used to treat various neurodegenerative diseases, such as Parkinson’s disease, Alzheimer’s disease, and amyotrophic lateral sclerosis. They have even been used with favorable results in animal models of acquired brain damage such as ischemia [11]. However, these extracts have not been used to treat patients with TBI or in experimental models of TBI. Due to the heterogeneity of traumatic brain injury, various clinical manifestations can develop from focal to diffuse damage in different brain regions, leading to temporary or permanent neurological and neuropsychiatric deficits [25]. Our results showed that mTBI increased the immobility duration in the forced swimming test from Day 7 to Day 60 post-TBI, consistent with TBI-induced depressive behavior.

This has been previously described in TBI models, including mTBI models [26]. Short-term administration of the lyophilized extract of the *M. pruriens* plant decreased the immobility duration to values similar to those of the control groups (the N and S groups). These behaviors were observed from Day 7 to Day 60.

Oxidative stress is one of the most important mechanisms of damage after injury to brain tissue because it generates a significant increase in pro-oxidant species in the surrounding tissue, which leads to massive neuronal death and, as a result, functional deficits [27]. Excessive generation of ROS and depletion of antioxidant defenses are essential contributors to the pathophysiology of secondary damage induced by TBI [28]. The generation of depressive disorders has been associated with alterations in neuronal plasticity and reduced volume of the frontal cortex, linked with an increase in the generation of ROS and depletion of antioxidant defenses [29]. Our findings indicate that the anti-depressant effect of treatment with the lyophilized extract of the *M. pruriens* plant (50 mg/kg, i.p.) in rats with mTBI may be modulated by decreases in ROS levels. Our data indicate that mTBI increased the LP concentration in the motor cortex, striatum, midbrain, and nucleus accumbens (significantly at 30 days post-TBI). Short-term administration of *M. pruriens* extract prevented this increase.

In contrast, reduced GSH values were not altered by mTBI in any of the regions analyzed; however, short-term administration of *M. pruriens* extract increased its levels in the motor cortex. According to previous reports, treatment with *M. pruriens* significantly improved spatial memory and reversed neuronal damage in ischemic rats, related to a decrease in ROS levels [30]. Therefore, the data obtained in our study suggest that the increase in GSH levels and the decrease in LP levels are linked to a decrease in ROS levels, leading to a decrease in depressive behaviors induced by brain damage. The potential antioxidant effect of the lyophilized extract of the *M. pruriens* plant may be responsible for the decrease in depressive behaviors (reduced immobility duration) since a decrease in lipid peroxidation was observed in the motor cortex, striatum nucleus, midbrain, and nucleus accumbens as pathways were not examined in this study, and reduced GSH concentrations increased in the motor cortex. These regions are predominantly associated with major depression in Parkinson’s disease [31]. However, more studies are needed to confirm this hypothesis.

## 5. Perspectives

Our results indicate that *M. pruriens* extract was able to prevent depression-like behavior induced by mTBI in a rat model. Likewise, it prevented lipid peroxidation in the midbrain and increased the levels of reduced GHS in the cerebral cortex. However, the administration scheme and doses did not reduce the sensory and motor impairment caused by mTBI, which was evaluated using the NSSR scale. Therefore, it would be necessary to analyze other doses and administration schemes. For example, administering *M. pruriens* extract before mTBI and/or administering it chronically.

On the other hand, it would be very interesting to analyze other mechanisms of action of the *M pruriens* extract in our mTBI model. For example, analyze if it affects the expression of proinflammatory cytokines such as TNFα. Furthermore, we still need to explore the effect of the isolated components of the extract used.
